# Supplementary figures and images for: DDX17 modulates the expression and alternative splicing of genes involved in apoptosis and proliferation in lung adenocarcinoma cells
Source: PeerJ. 2022 Sep 21;10:e13895. doi: 10.7717/peerj.13895 (PMC9508879; doi:10.7717/peerj.13895)

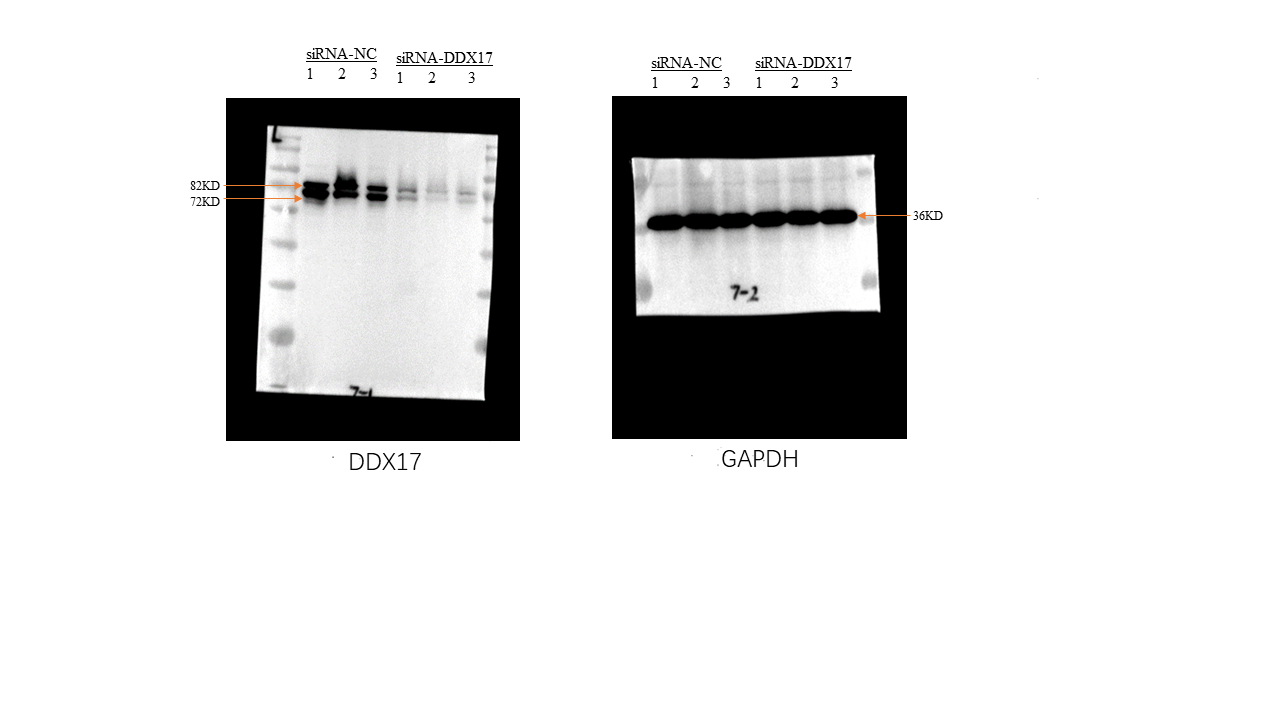

Supplement: Supplemental Information 2 — The efficiency of DDX17 siRNA knockdown was measured by western blot. Uncropped original blots are shown. [file peerj-10-13895-s002.png]

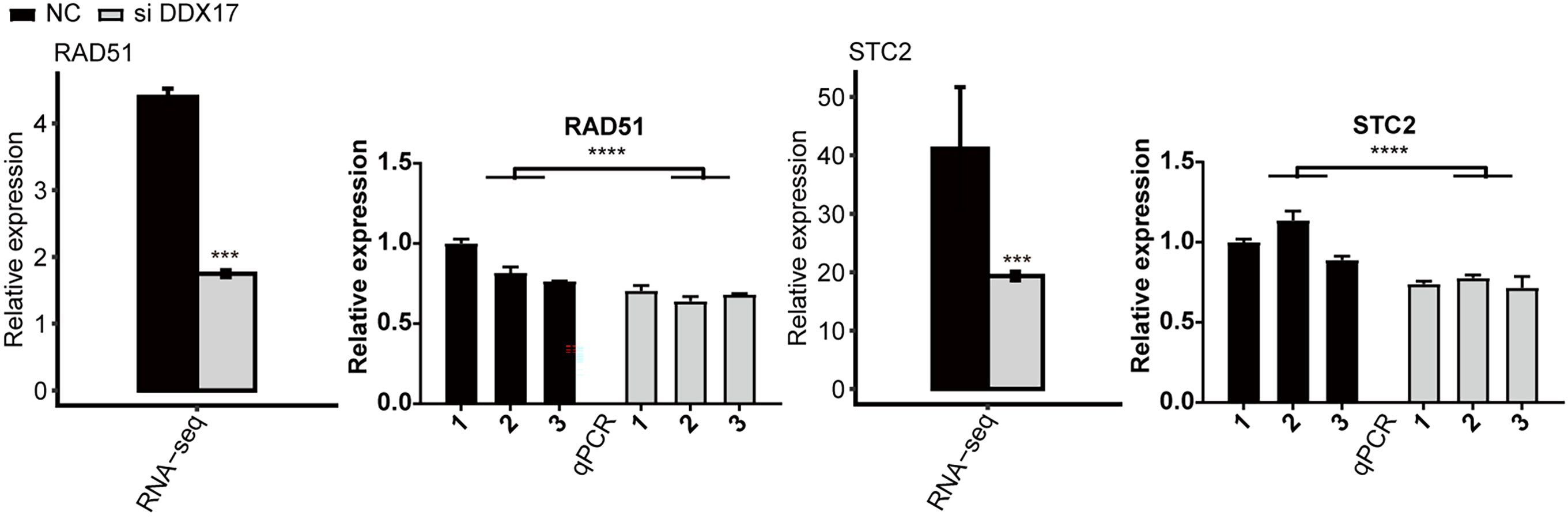

Supplement: Supplemental Information 3 — The error bars indicate the means ± SEMs. ∗∗∗p < 0.001. ∗∗∗∗p < 0.0001. [file peerj-10-13895-s003.png]

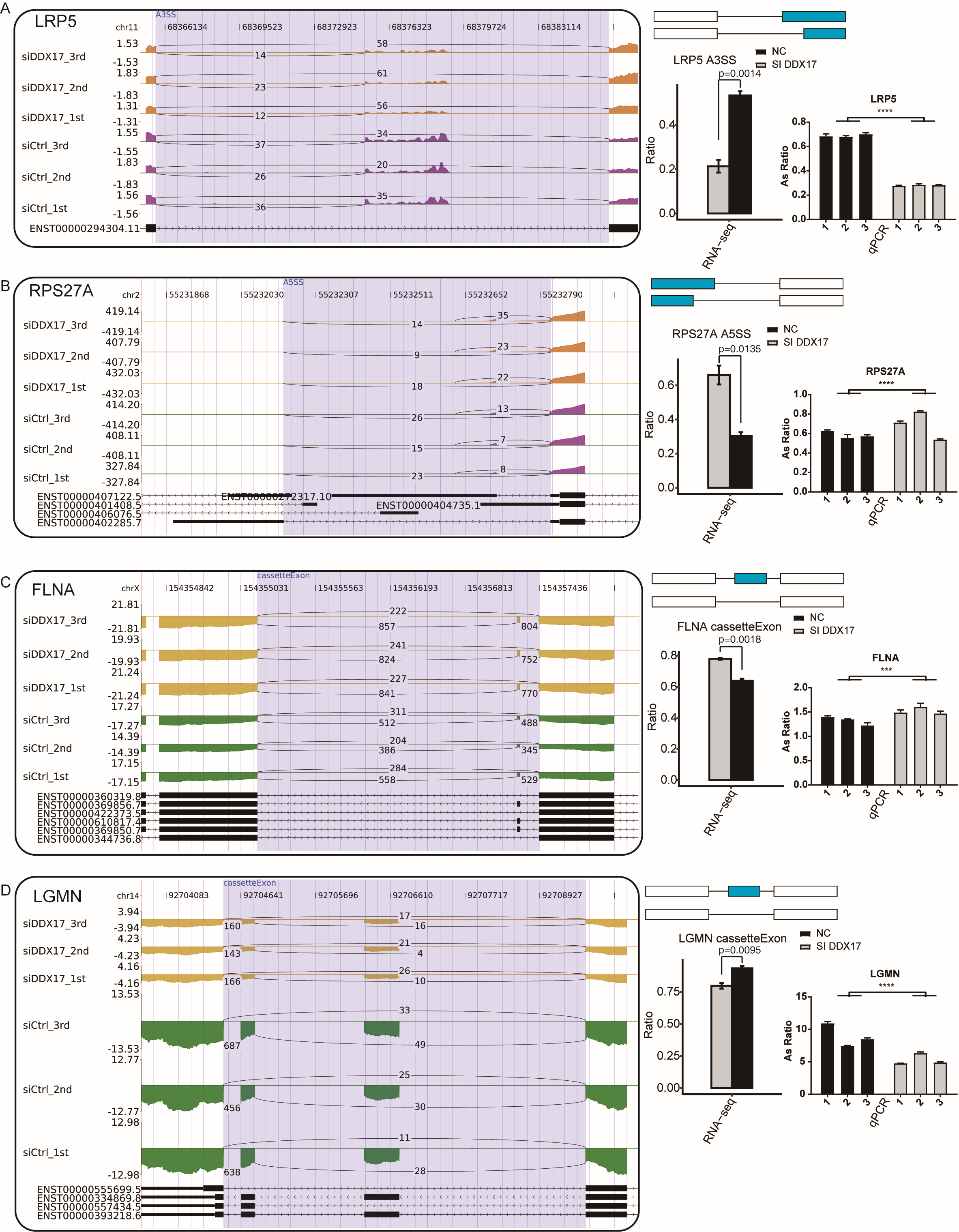

Supplement: Supplemental Information 4 — (A–D) IGV-sashimi plots show AS changes in DDX17 knockdown cells and control cells (left panel), and the transcripts for the gene are shown below. The schematic diagrams depict the structures of ASEs (right panel, top). The constitutive exon sequences are denoted with white boxes, intron sequences with horizontal line, while alternative exons with blue boxes. RNA-seq quantification and RT-qPCR validation of ASEs are shown at the bottom of the right panel. The altered ratio of AS events in RNA-seq was calculated using the formula: alternative splice junction reads/(alternative splice junction reads + model splice junction reads). Student’s t test was performed to compare DDX17-KD and control cells with significance set at a P value of less than 0.05. [file peerj-10-13895-s004.png]

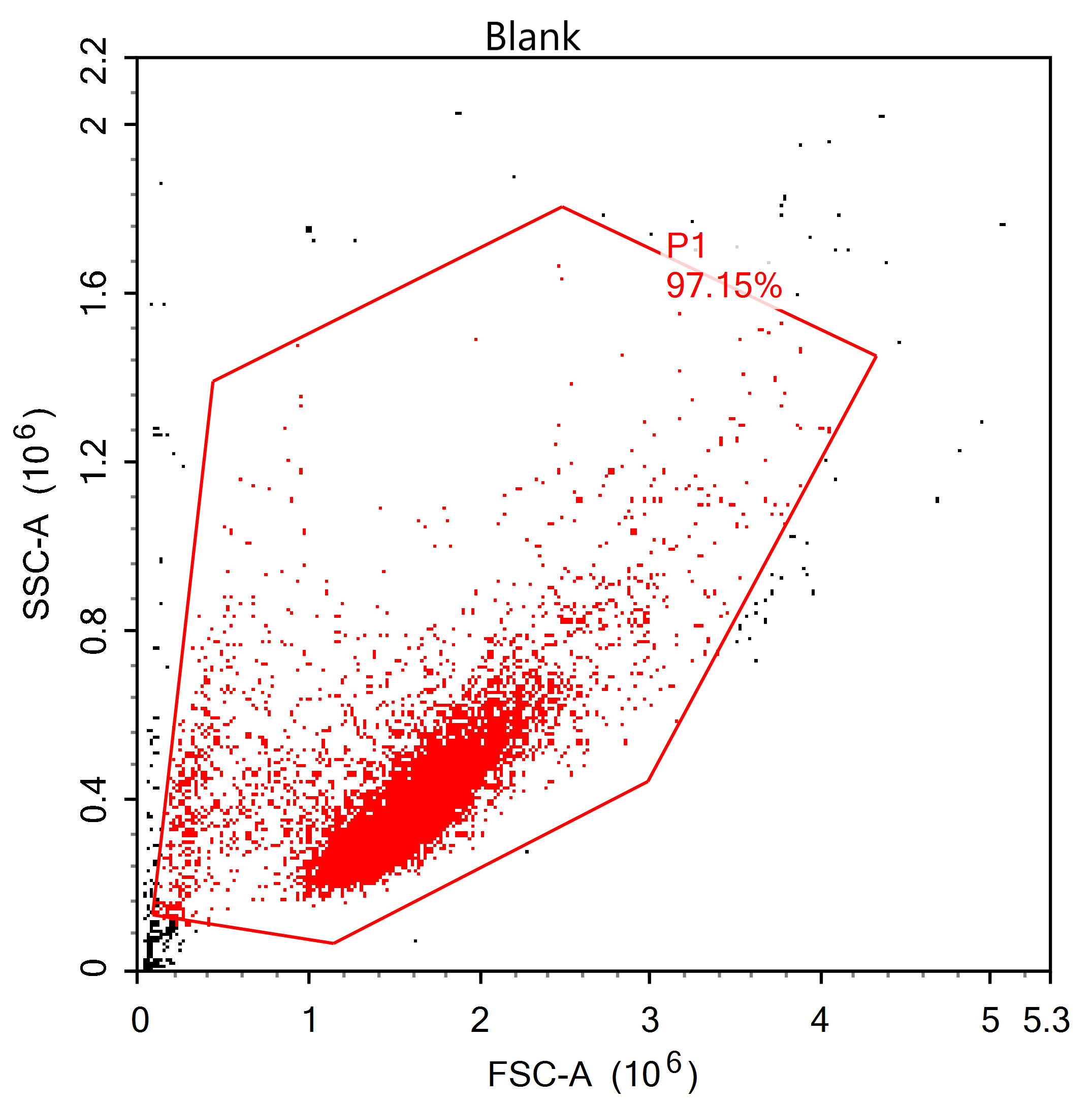

Supplement: Supplemental Information 6 [file peerj-10-13895-s006.zip › supplementary file 4 raw data of cell proliferation and apoptosis/apoptosis/Figures/Blank/Figure 1.tiff]

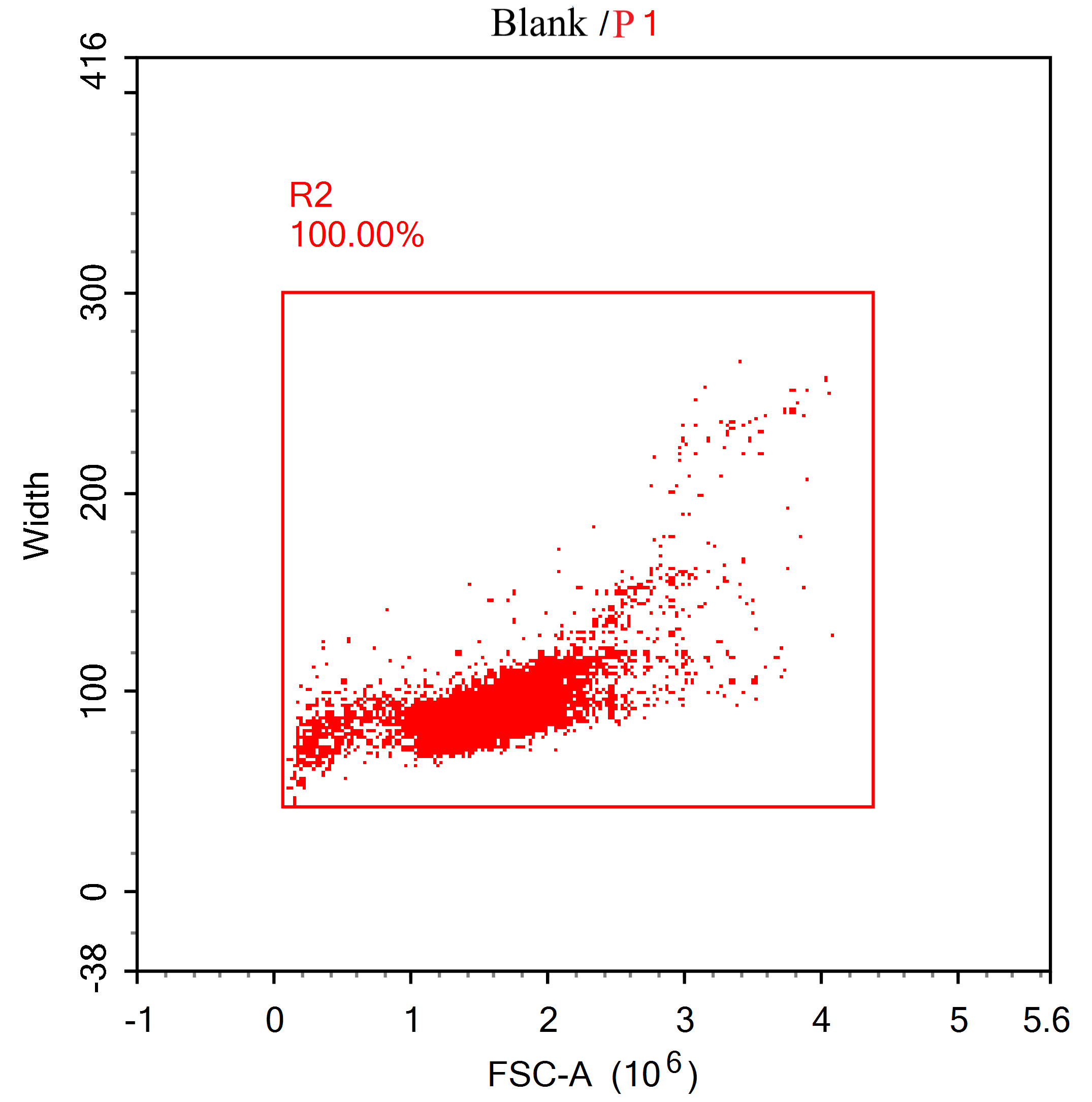

Supplement: Supplemental Information 6 [file peerj-10-13895-s006.zip › supplementary file 4 raw data of cell proliferation and apoptosis/apoptosis/Figures/Blank/Figure 2.tiff]

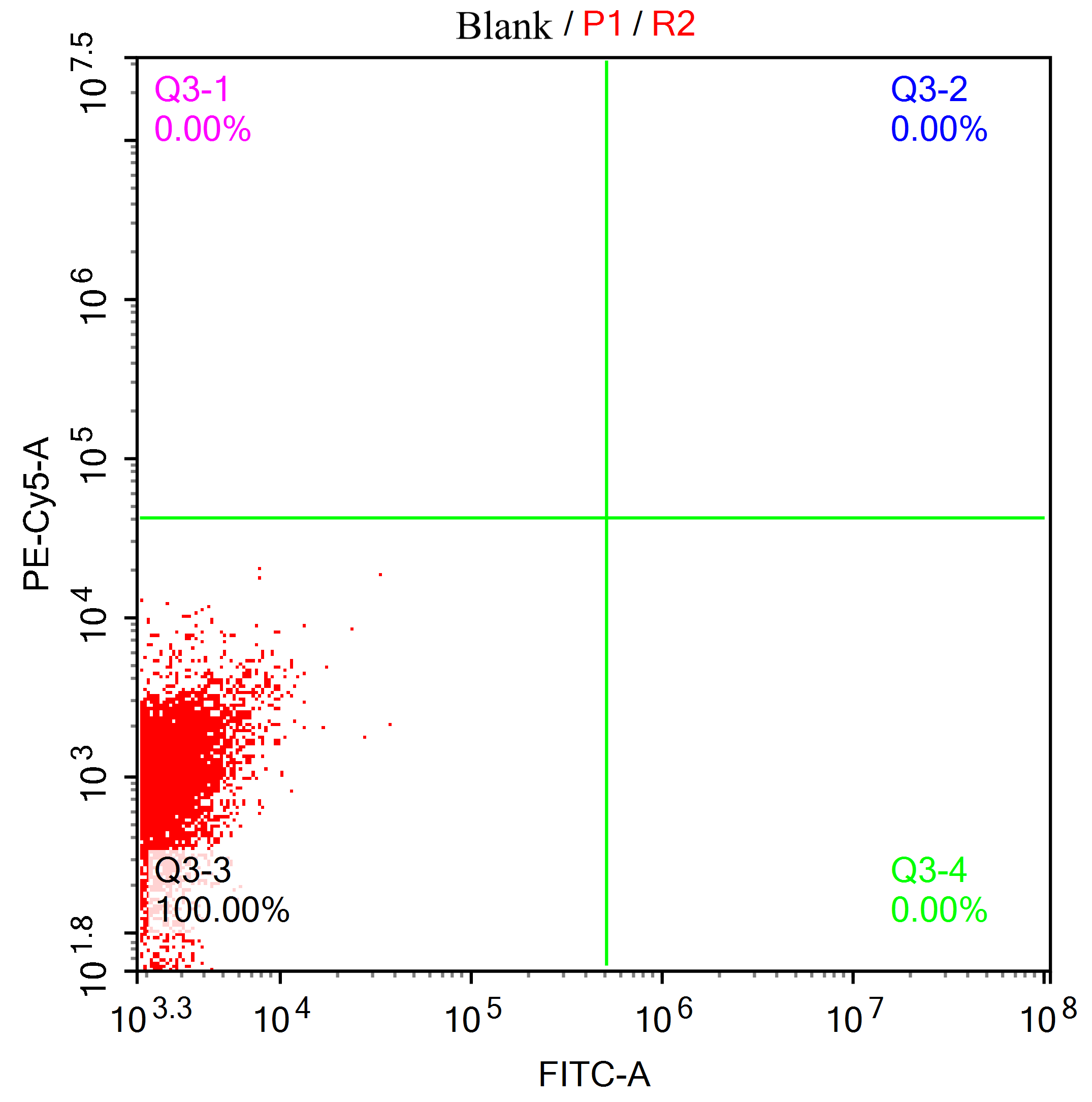

Supplement: Supplemental Information 6 [file peerj-10-13895-s006.zip › supplementary file 4 raw data of cell proliferation and apoptosis/apoptosis/Figures/Blank/Figure 3.tiff]

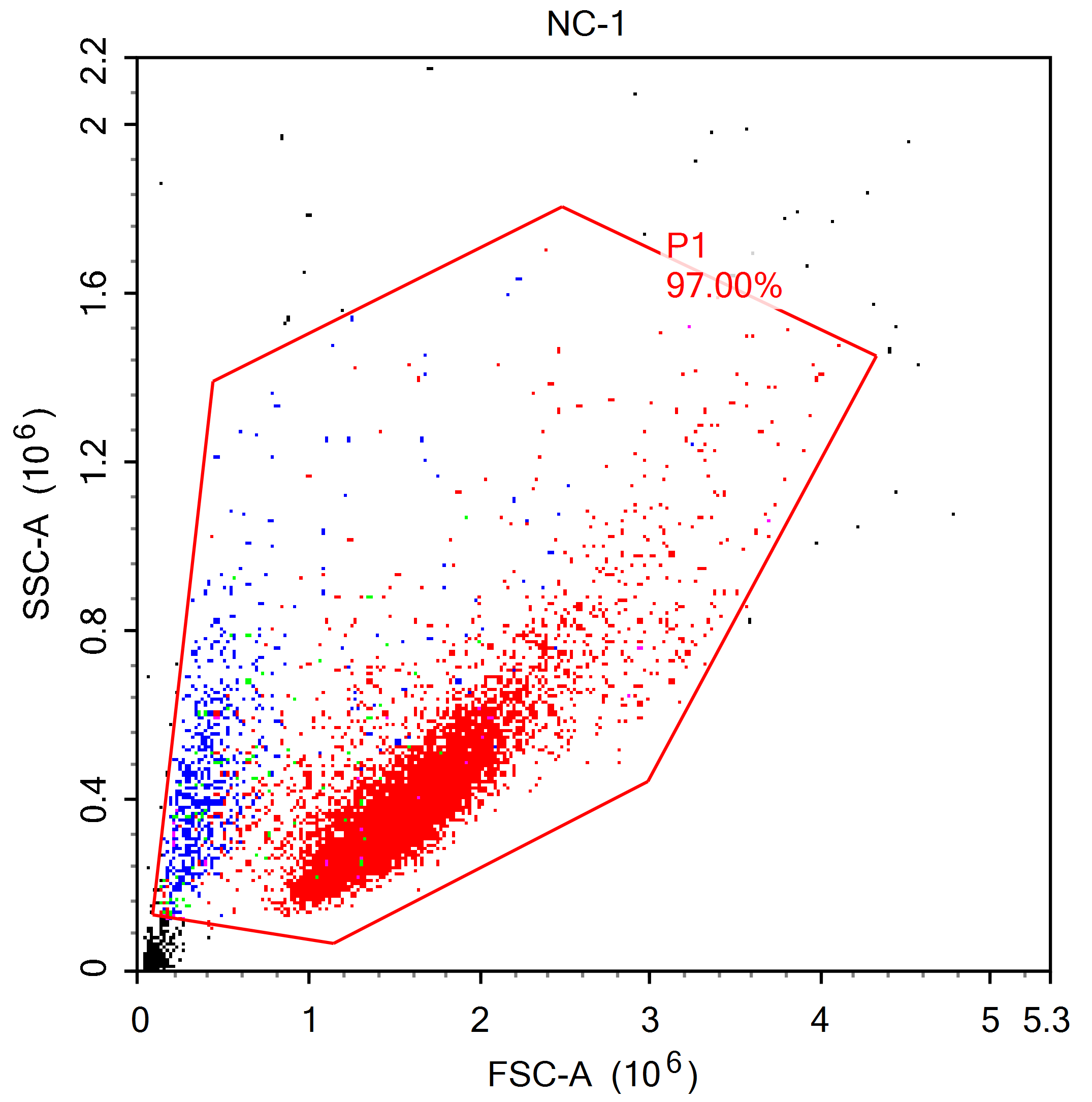

Supplement: Supplemental Information 6 [file peerj-10-13895-s006.zip › supplementary file 4 raw data of cell proliferation and apoptosis/apoptosis/Figures/NC-1/Figure 1.tiff]

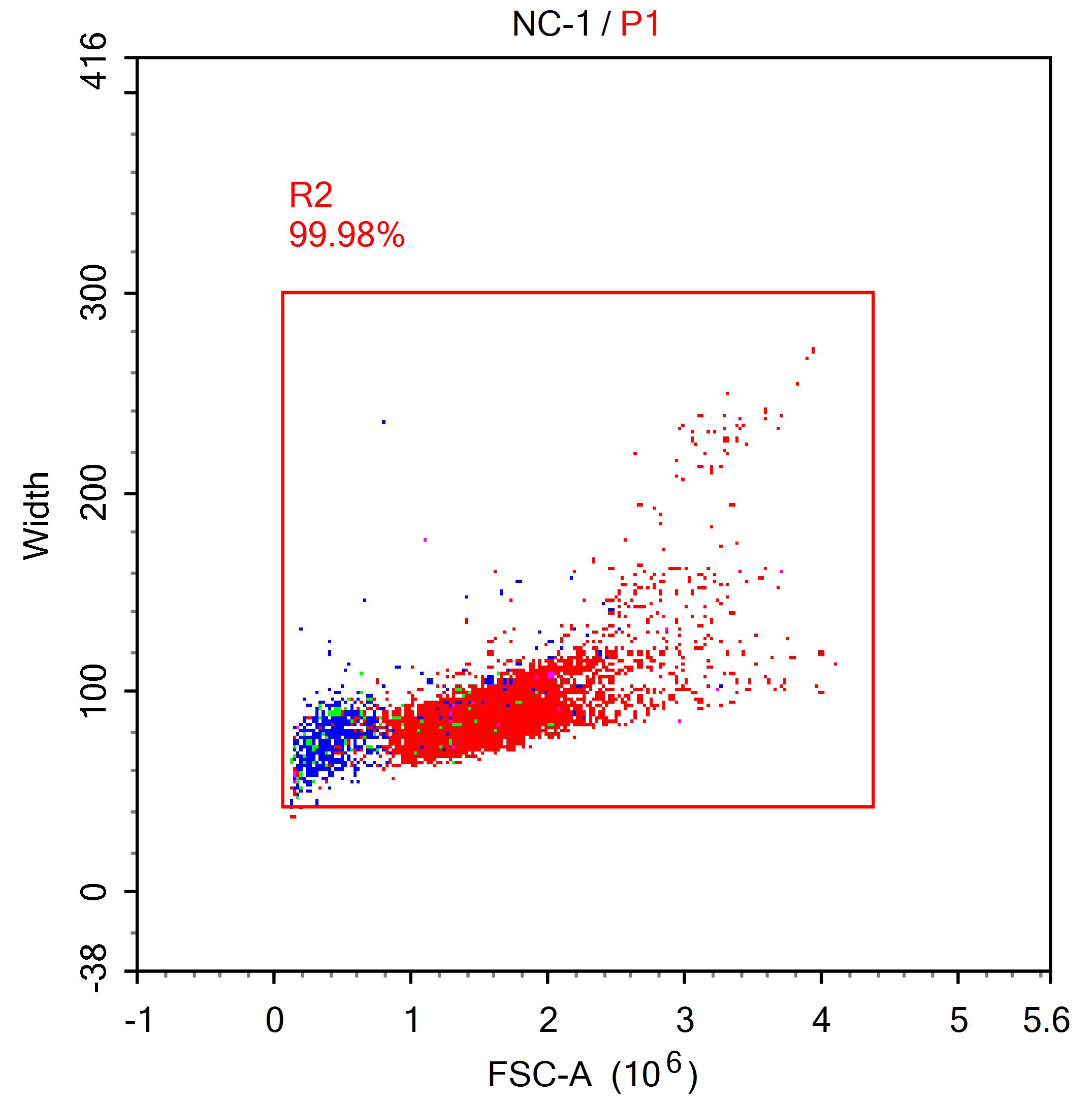

Supplement: Supplemental Information 6 [file peerj-10-13895-s006.zip › supplementary file 4 raw data of cell proliferation and apoptosis/apoptosis/Figures/NC-1/Figure 2.tiff]

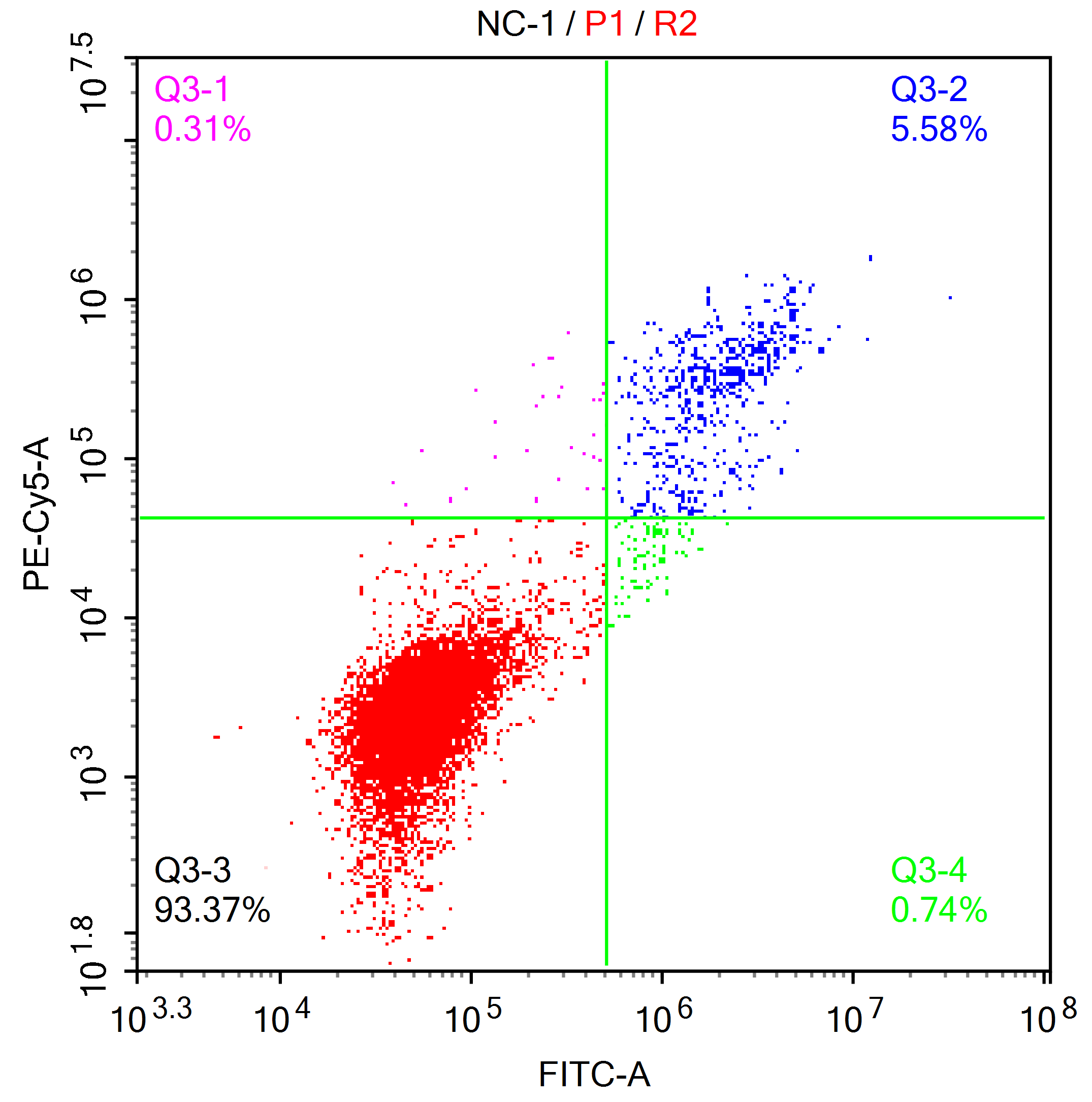

Supplement: Supplemental Information 6 [file peerj-10-13895-s006.zip › supplementary file 4 raw data of cell proliferation and apoptosis/apoptosis/Figures/NC-1/Figure 3.tiff]

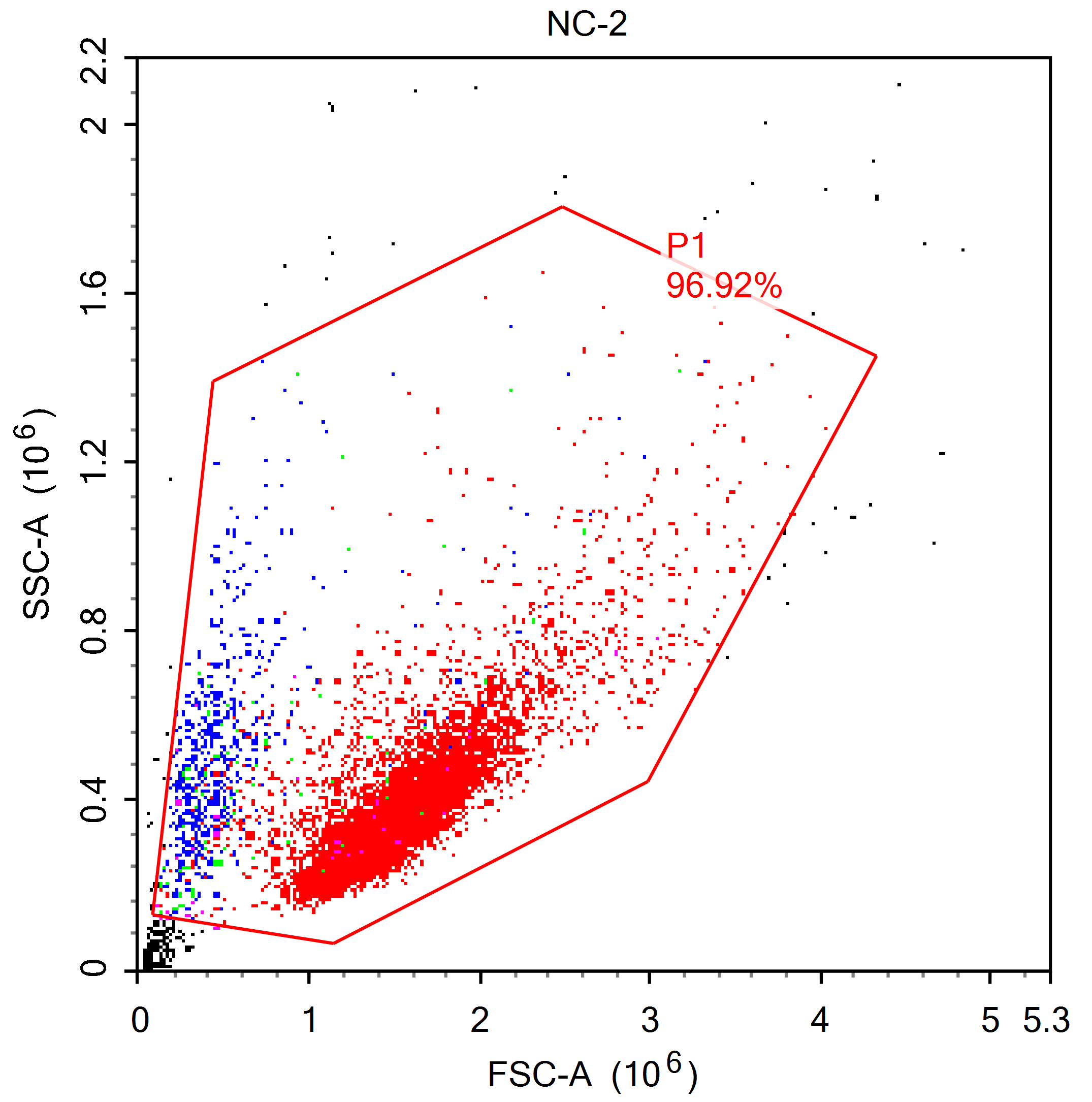

Supplement: Supplemental Information 6 [file peerj-10-13895-s006.zip › supplementary file 4 raw data of cell proliferation and apoptosis/apoptosis/Figures/NC-2/Figure 1.tiff]

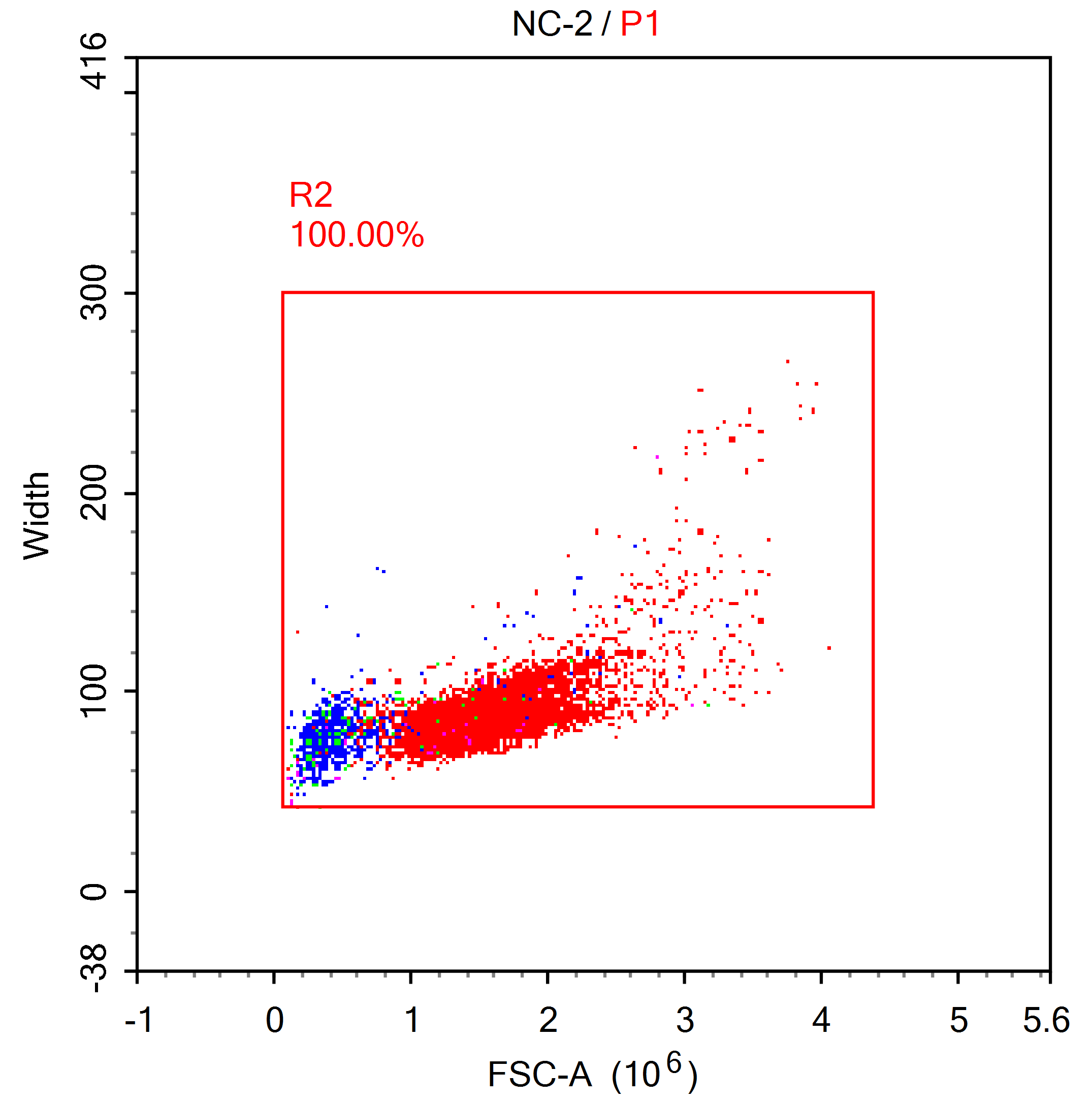

Supplement: Supplemental Information 6 [file peerj-10-13895-s006.zip › supplementary file 4 raw data of cell proliferation and apoptosis/apoptosis/Figures/NC-2/Figure 2.tiff]

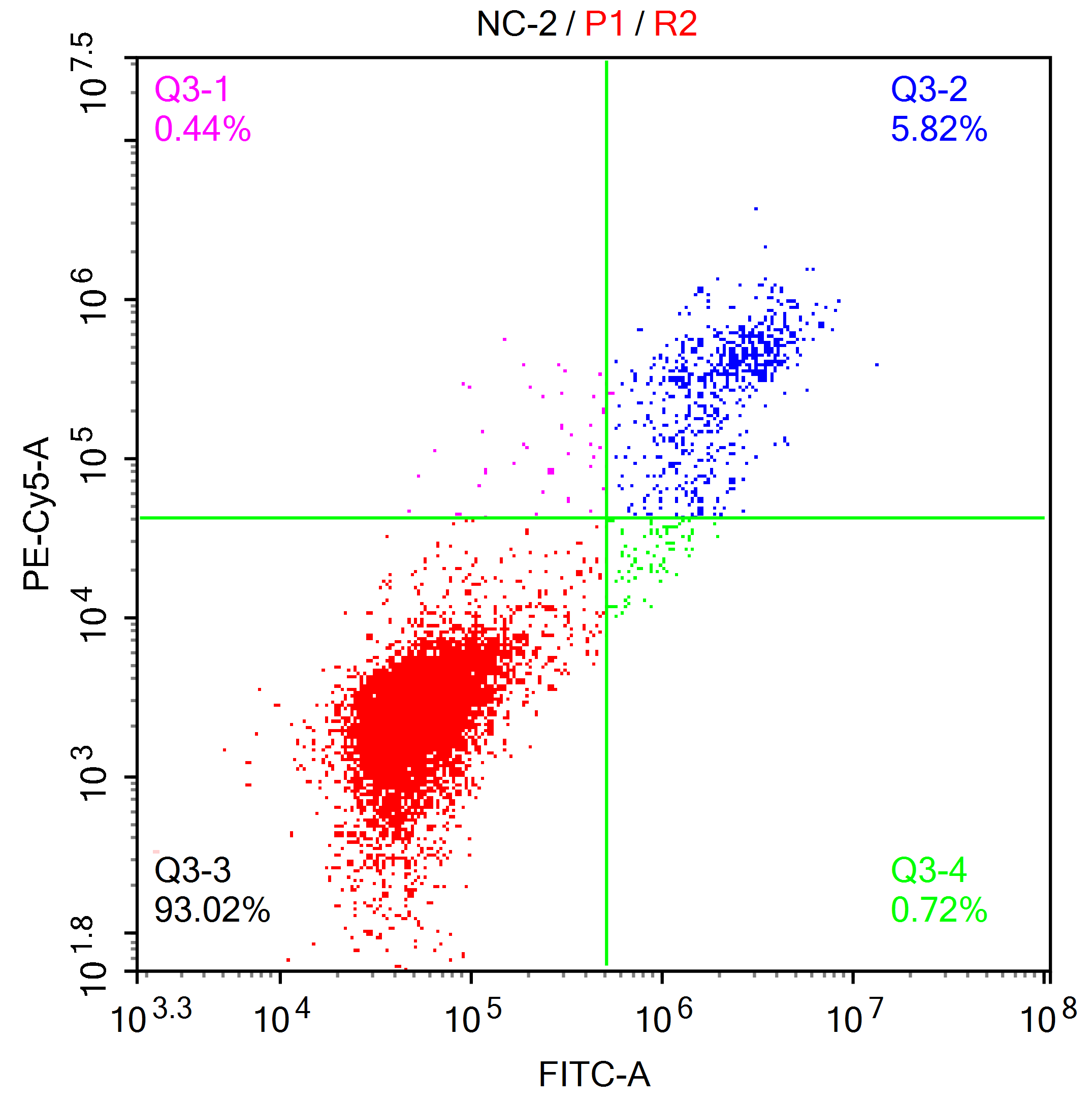

Supplement: Supplemental Information 6 [file peerj-10-13895-s006.zip › supplementary file 4 raw data of cell proliferation and apoptosis/apoptosis/Figures/NC-2/Figure 3.tiff]

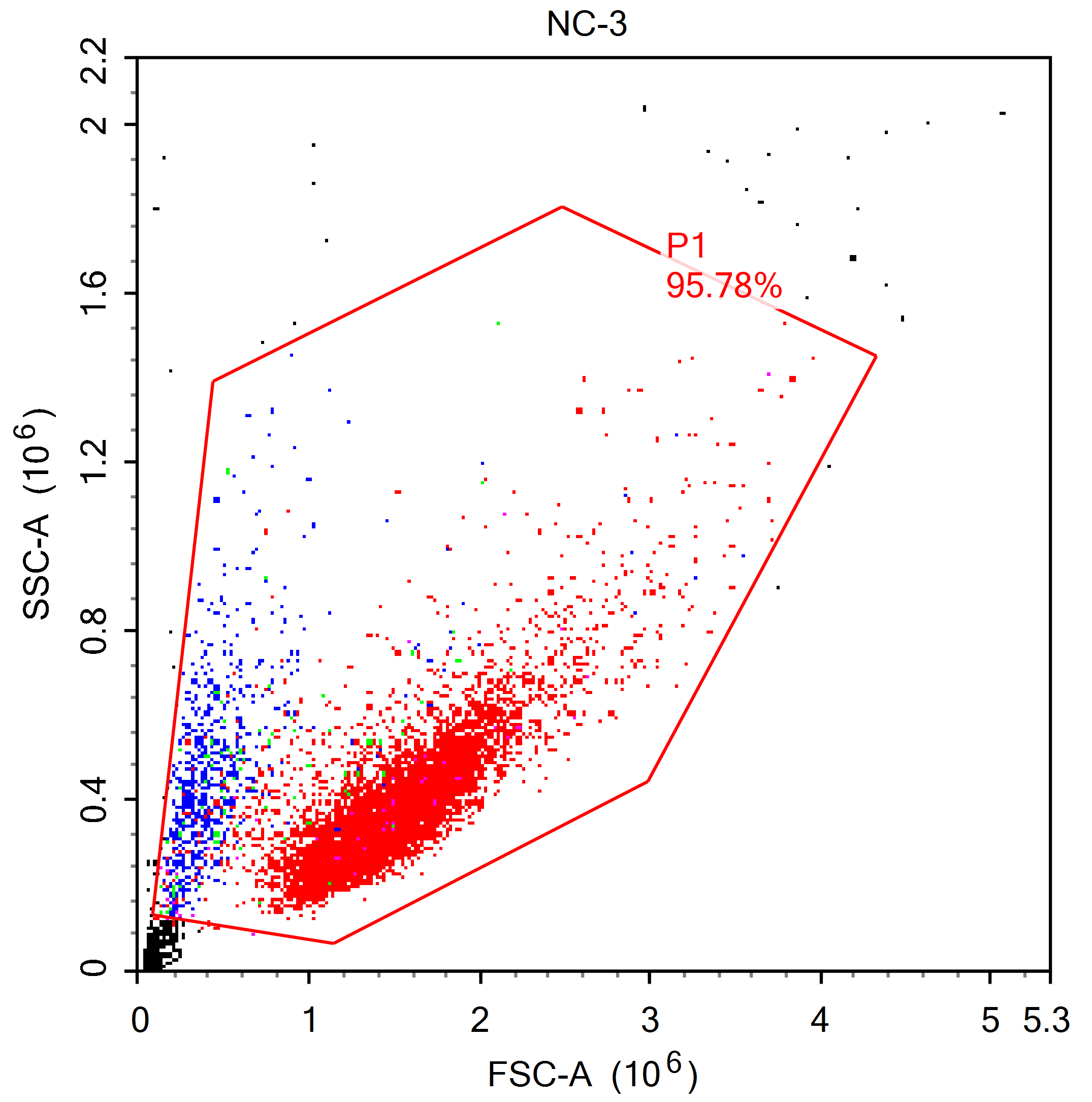

Supplement: Supplemental Information 6 [file peerj-10-13895-s006.zip › supplementary file 4 raw data of cell proliferation and apoptosis/apoptosis/Figures/NC-3/Figure 1.tiff]

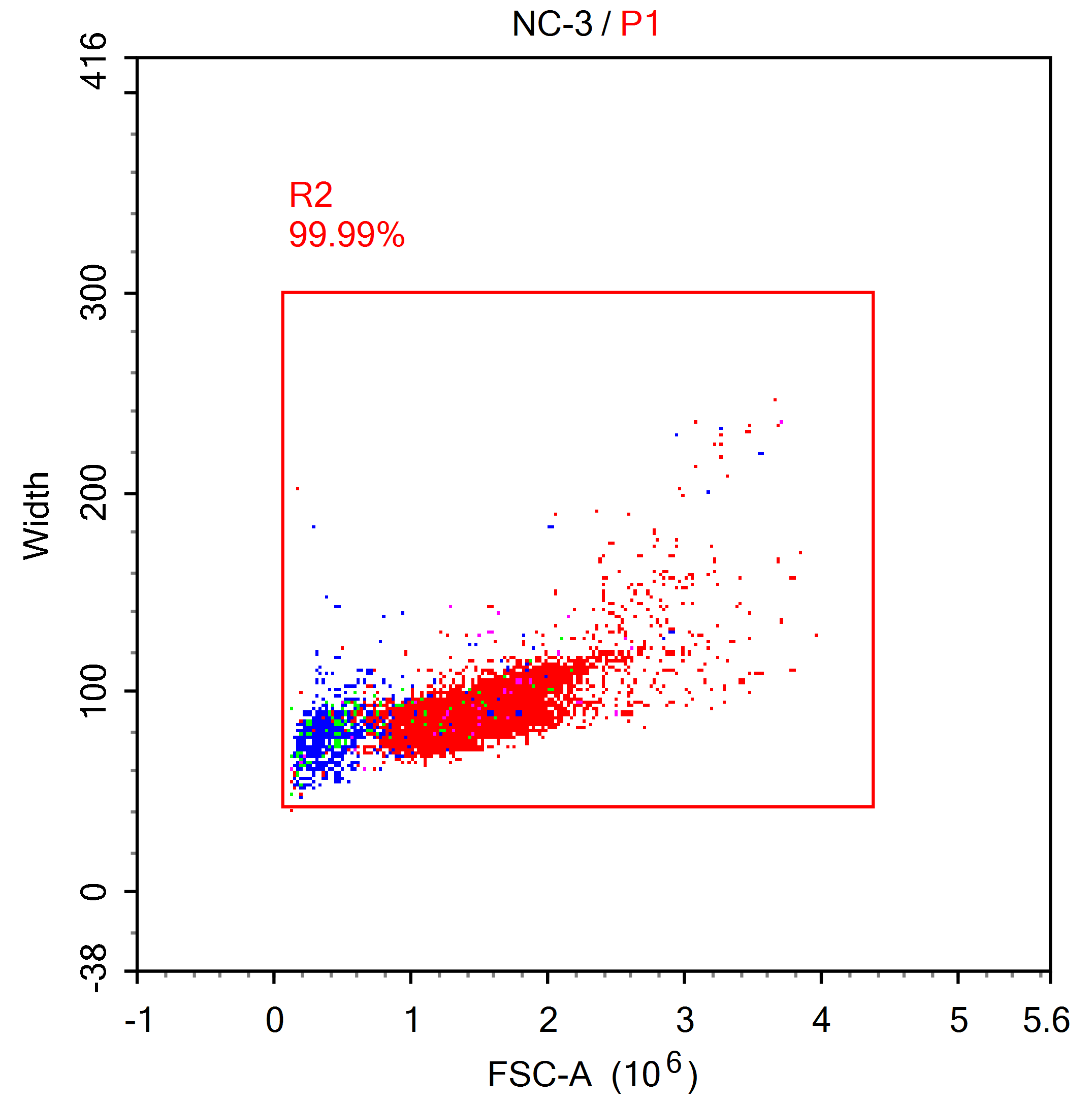

Supplement: Supplemental Information 6 [file peerj-10-13895-s006.zip › supplementary file 4 raw data of cell proliferation and apoptosis/apoptosis/Figures/NC-3/Figure 2.tiff]

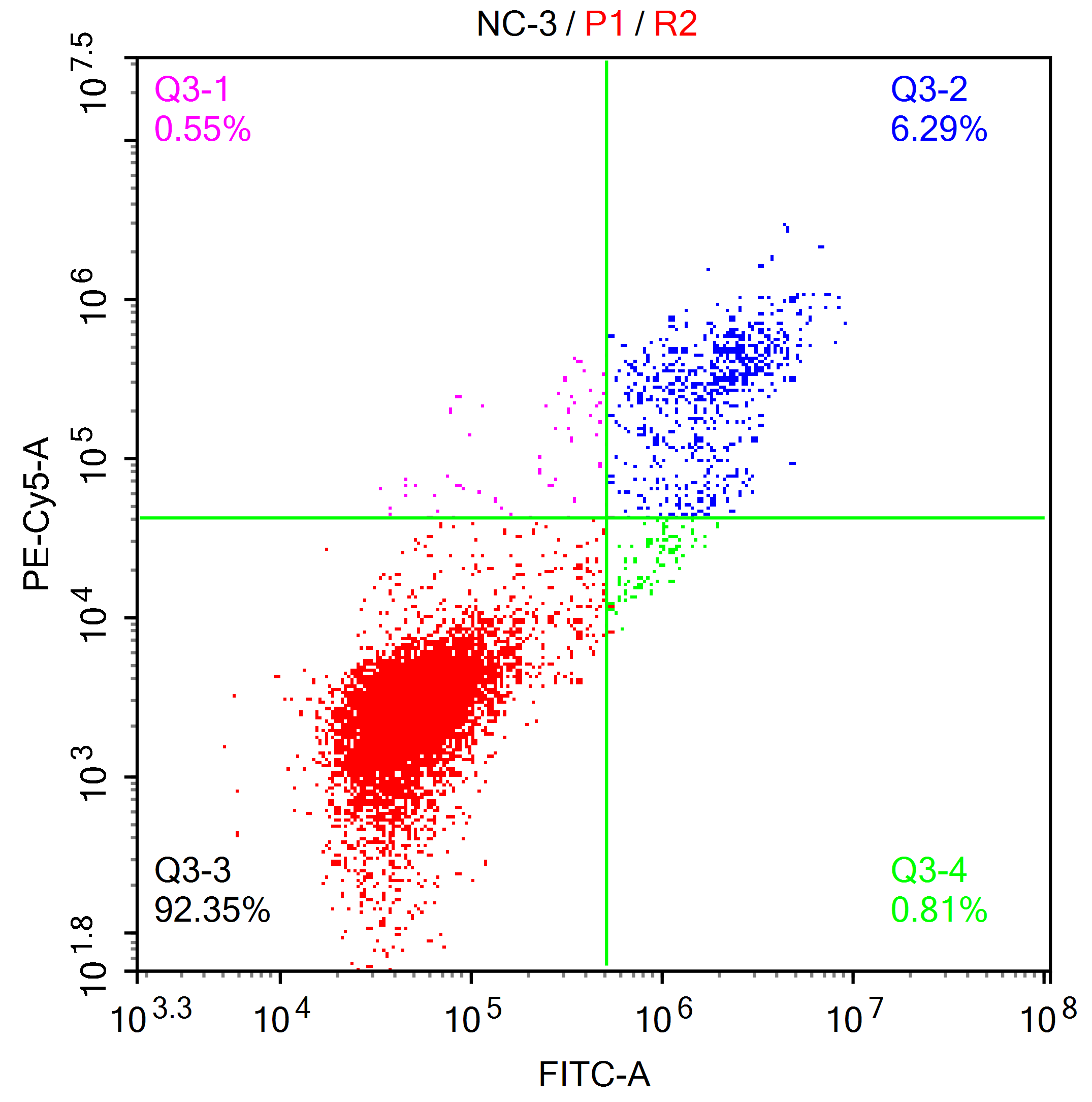

Supplement: Supplemental Information 6 [file peerj-10-13895-s006.zip › supplementary file 4 raw data of cell proliferation and apoptosis/apoptosis/Figures/NC-3/Figure 3.tiff]

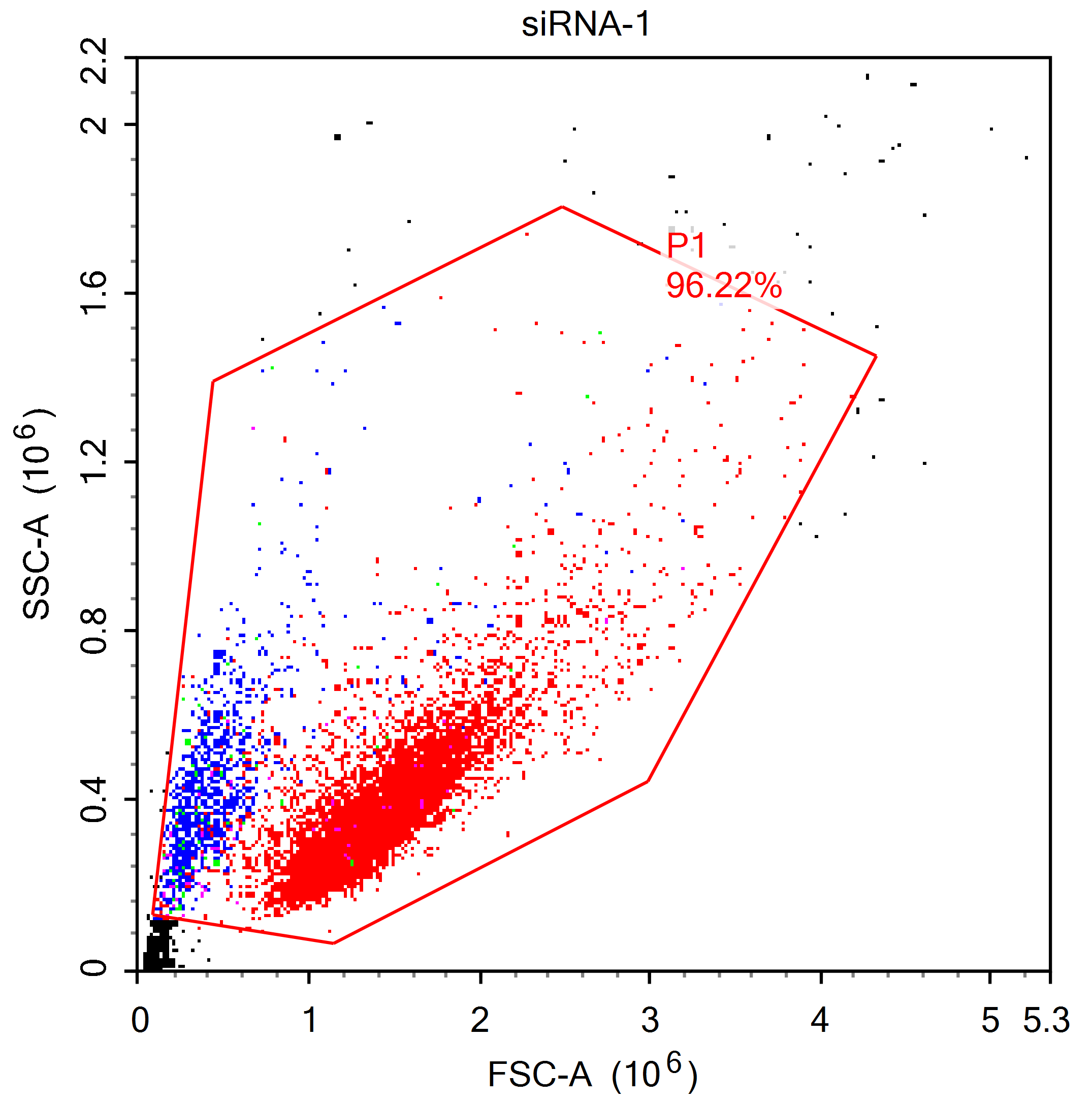

Supplement: Supplemental Information 6 [file peerj-10-13895-s006.zip › supplementary file 4 raw data of cell proliferation and apoptosis/apoptosis/Figures/siRNA-1/Figure 1.tiff]

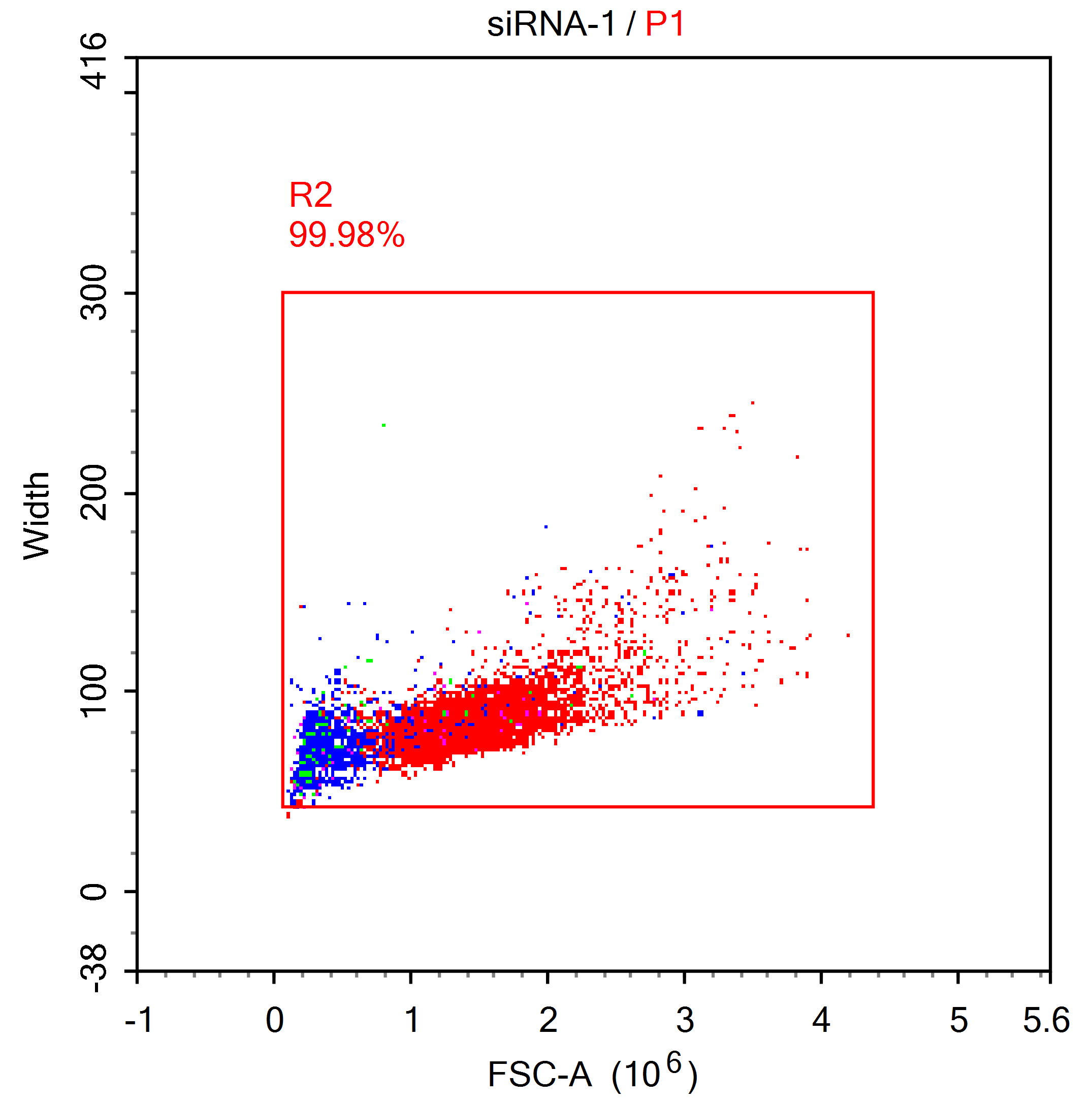

Supplement: Supplemental Information 6 [file peerj-10-13895-s006.zip › supplementary file 4 raw data of cell proliferation and apoptosis/apoptosis/Figures/siRNA-1/Figure 2.tiff]

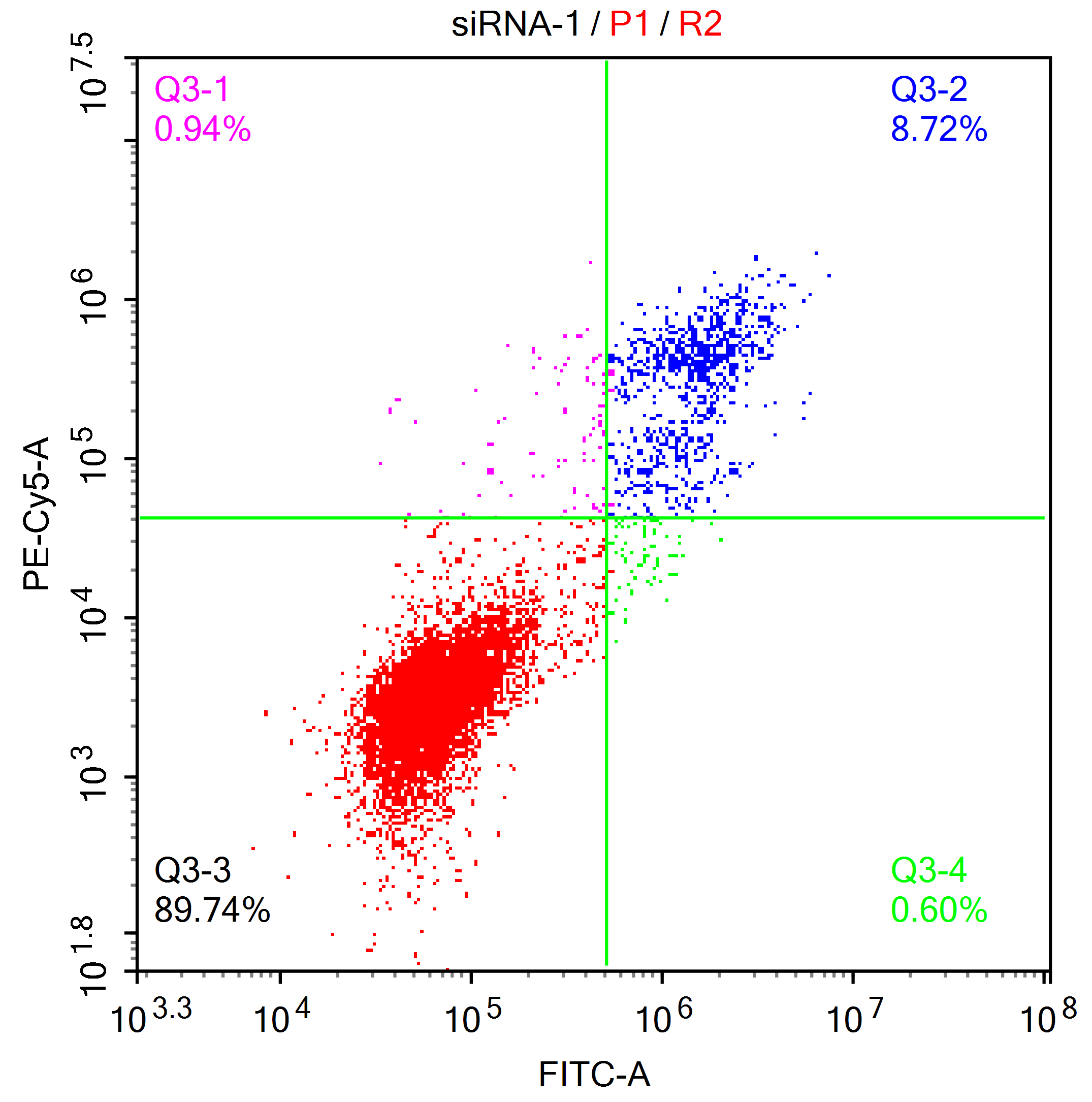

Supplement: Supplemental Information 6 [file peerj-10-13895-s006.zip › supplementary file 4 raw data of cell proliferation and apoptosis/apoptosis/Figures/siRNA-1/Figure 3.tiff]

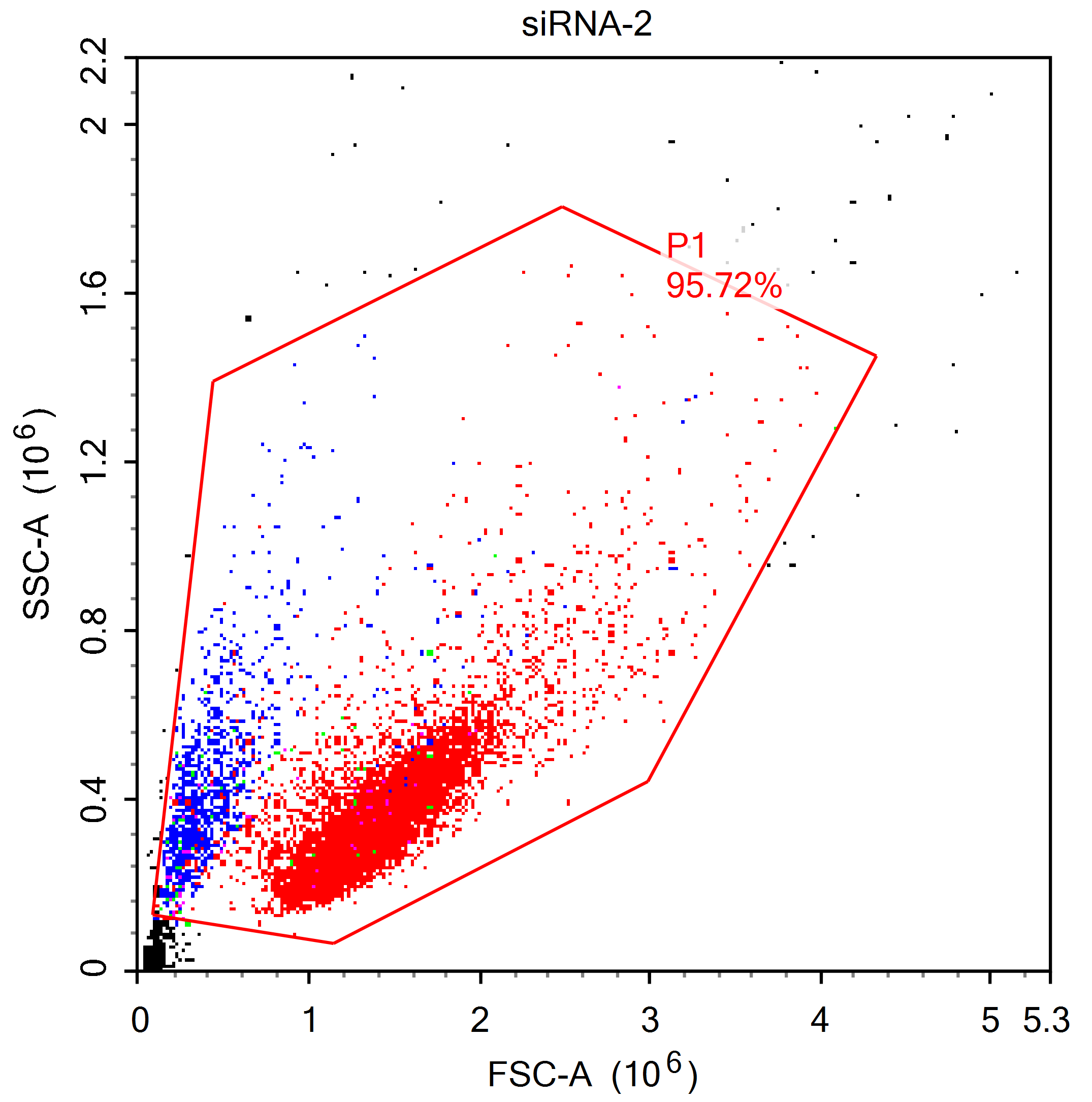

Supplement: Supplemental Information 6 [file peerj-10-13895-s006.zip › supplementary file 4 raw data of cell proliferation and apoptosis/apoptosis/Figures/siRNA-2/Figure 1.tiff]

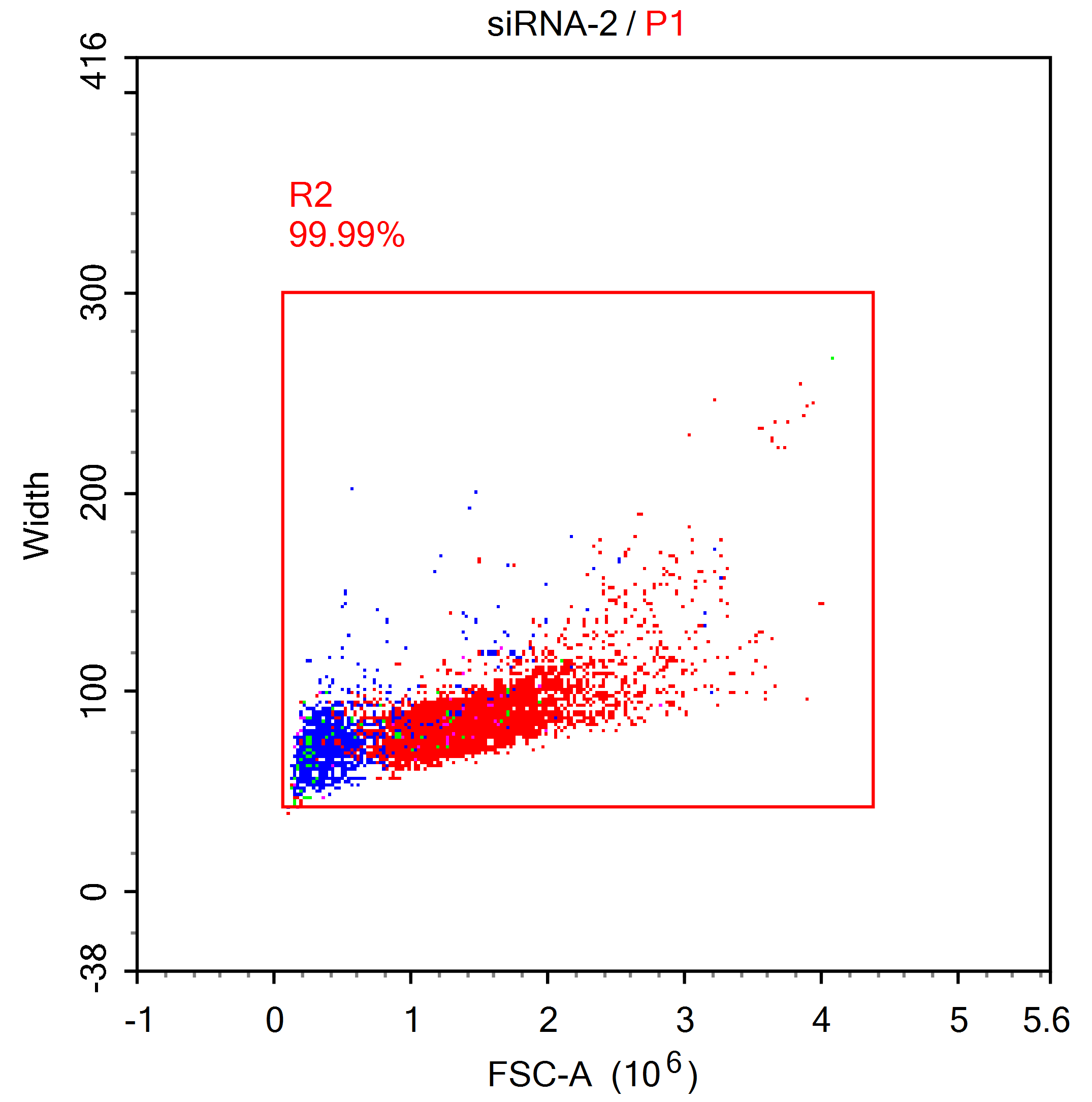

Supplement: Supplemental Information 6 [file peerj-10-13895-s006.zip › supplementary file 4 raw data of cell proliferation and apoptosis/apoptosis/Figures/siRNA-2/Figure 2.tiff]

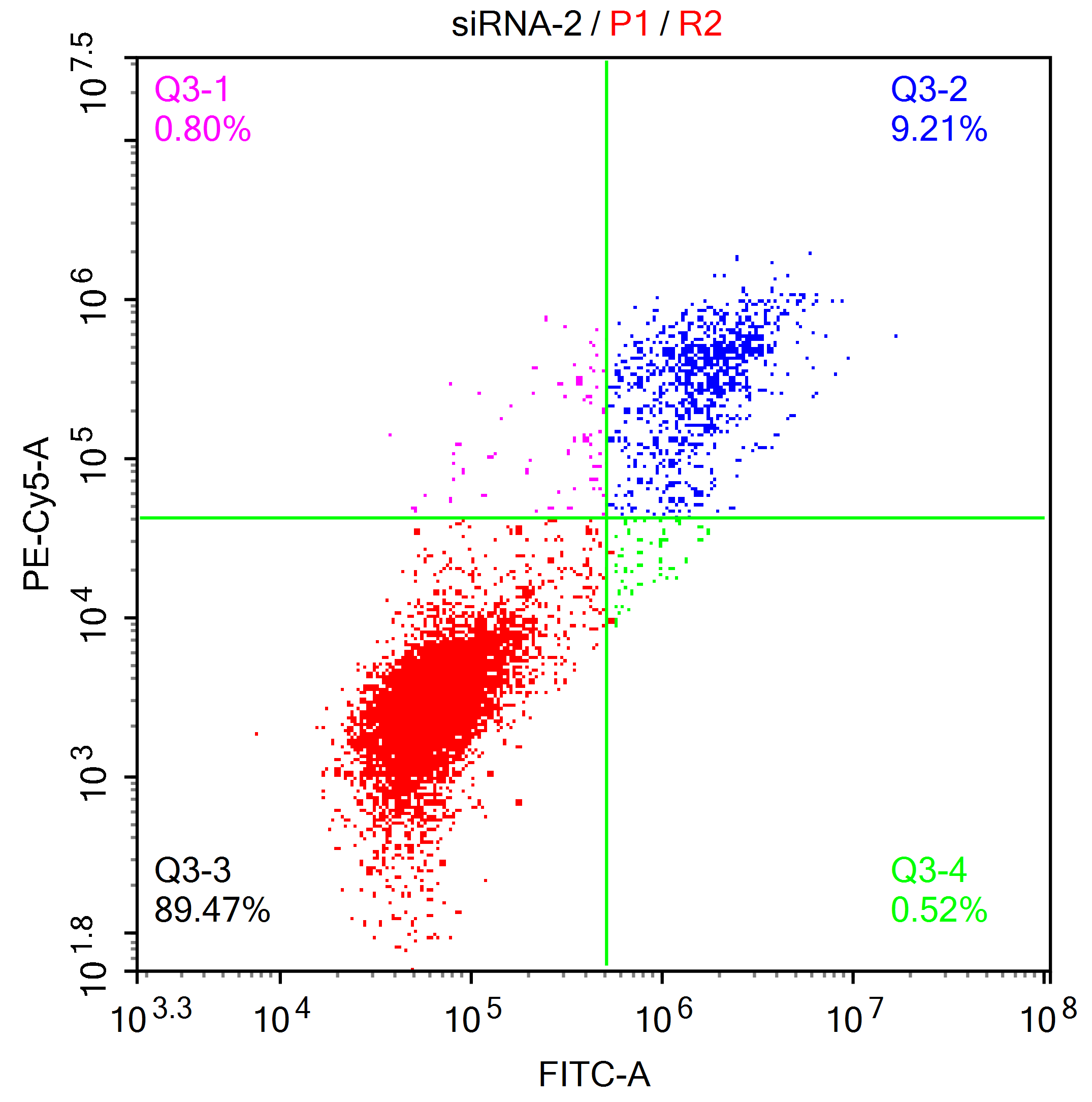

Supplement: Supplemental Information 6 [file peerj-10-13895-s006.zip › supplementary file 4 raw data of cell proliferation and apoptosis/apoptosis/Figures/siRNA-2/Figure 3.tiff]

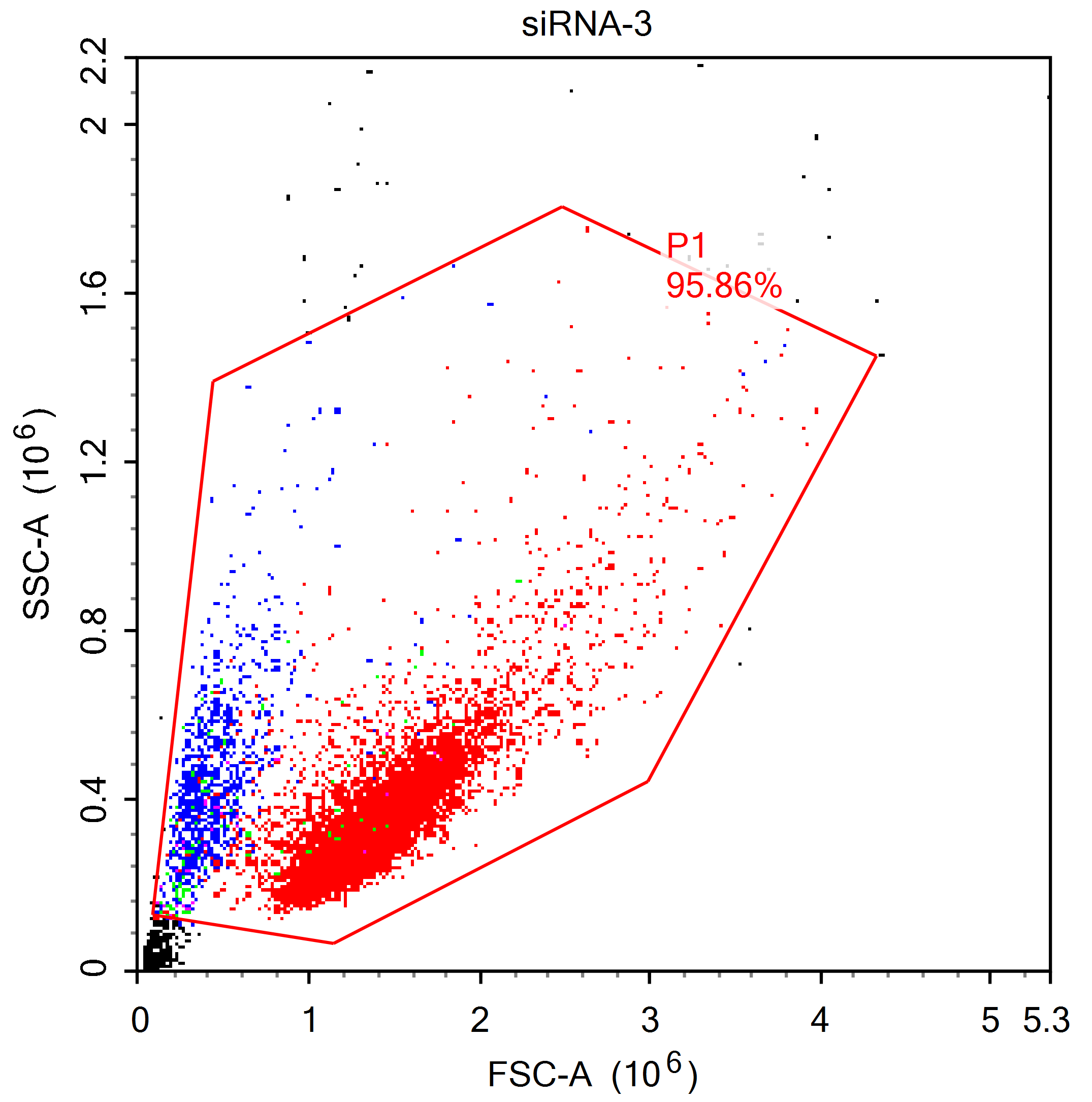

Supplement: Supplemental Information 6 [file peerj-10-13895-s006.zip › supplementary file 4 raw data of cell proliferation and apoptosis/apoptosis/Figures/siRNA-3/Figure 1.tiff]

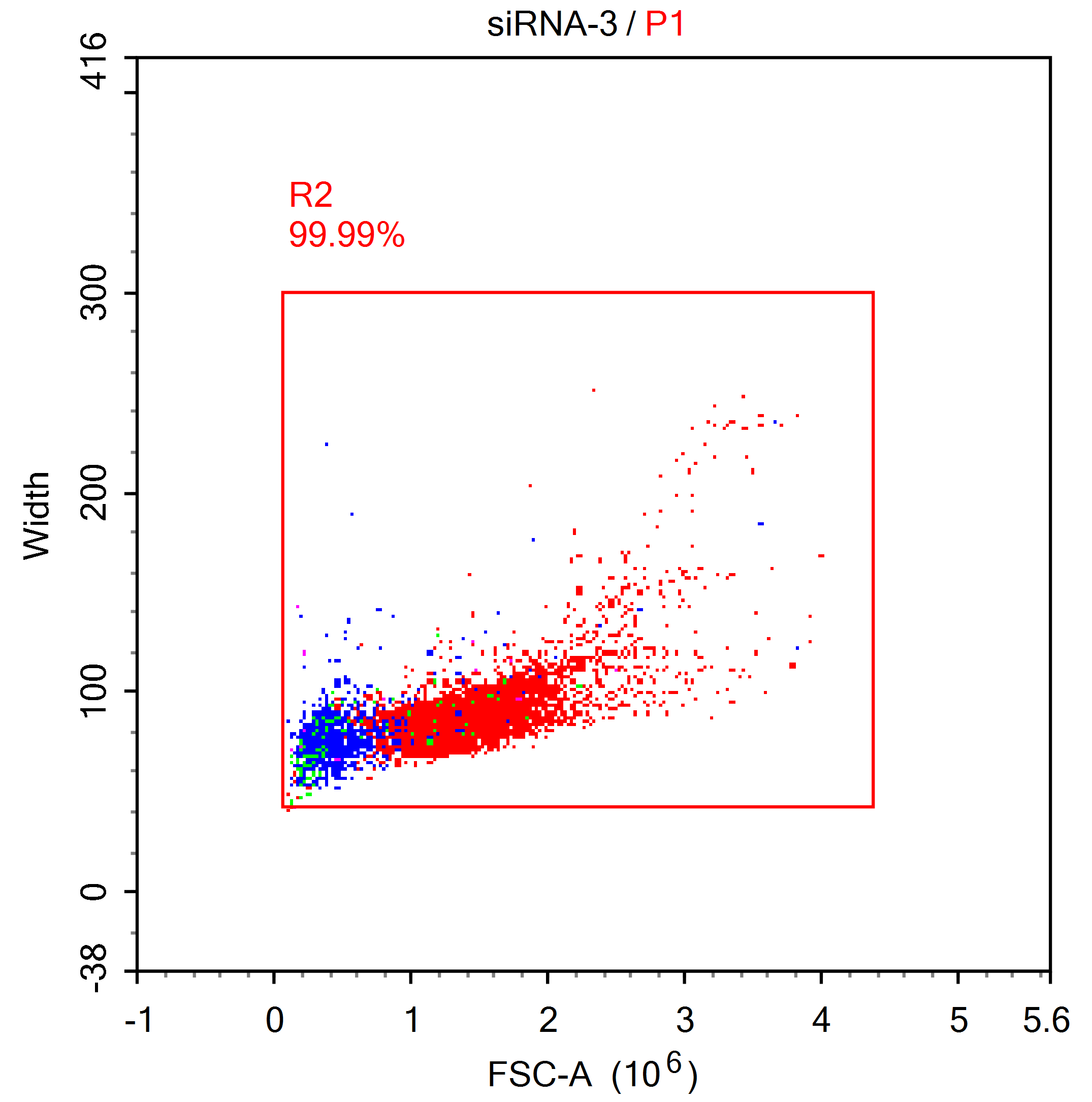

Supplement: Supplemental Information 6 [file peerj-10-13895-s006.zip › supplementary file 4 raw data of cell proliferation and apoptosis/apoptosis/Figures/siRNA-3/Figure 2.tiff]

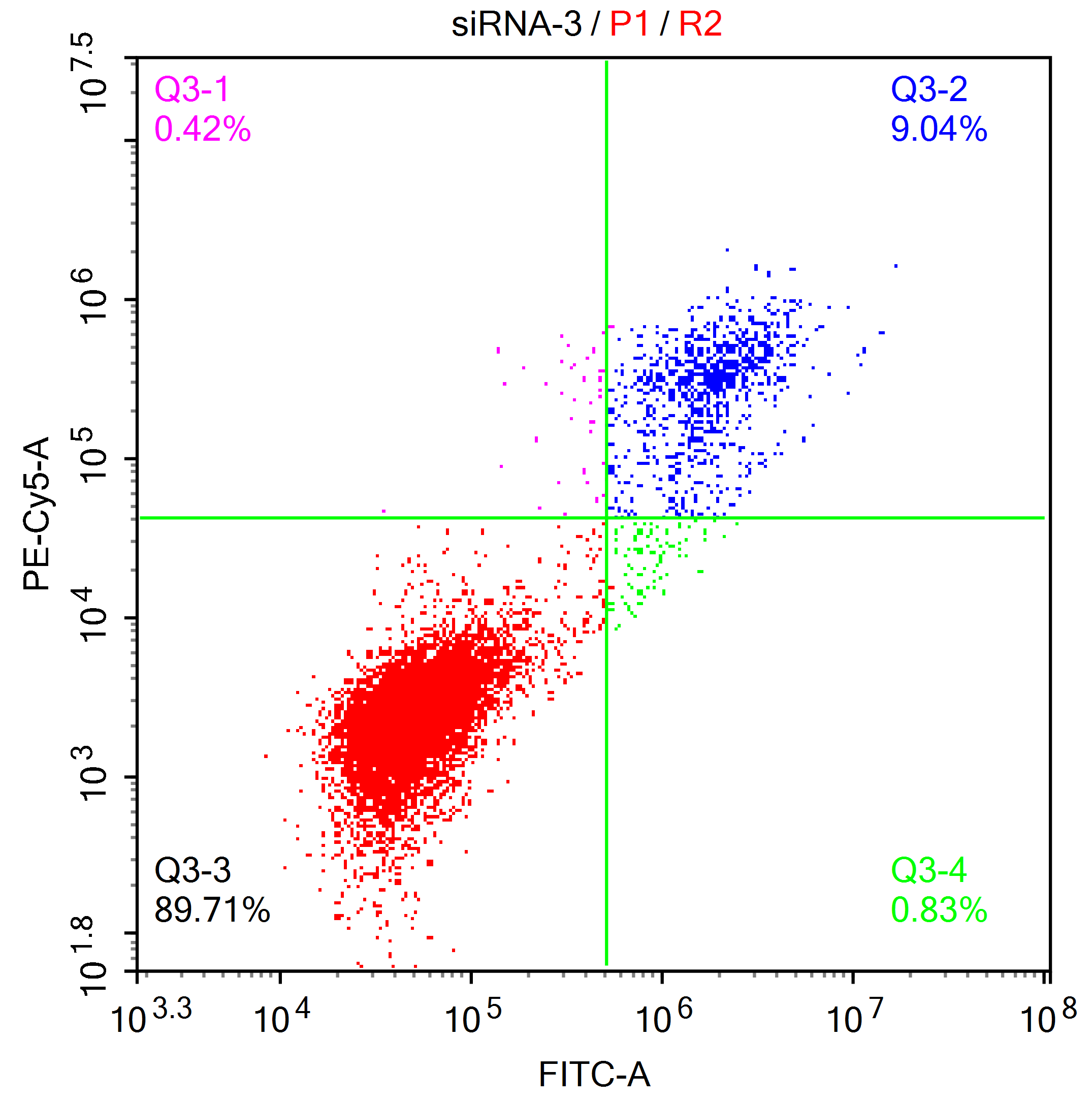

Supplement: Supplemental Information 6 [file peerj-10-13895-s006.zip › supplementary file 4 raw data of cell proliferation and apoptosis/apoptosis/Figures/siRNA-3/Figure 3.tiff]
