# Supplementary material for: DDX17 modulates the expression and alternative splicing of genes involved in apoptosis and proliferation in lung adenocarcinoma cells
Source: PeerJ. 2022 Sep 21;10:e13895. doi: 10.7717/peerj.13895 (PMC9508879; doi:10.7717/peerj.13895)
Supplement: Supplemental Information 7 [file peerj-10-13895-s007.zip › raw data of qPCR/primer Information for AS and DEG.docx]

| RAD51-F | GGAGACAGGTCAGTAGTCACAA |
| --- | --- |
| RAD51-R | CAAGTCATTCCTAAGGCACCAT |
| TSG101-F | GGAAGACATCCAGGTCTATCAC |
| TSG101-R | CCACAGCTCCCTTATACAAACA |
| LGMN-F | CAGGTAACAGCGAGCAAGTC |
| LGMN-R | GGAGAGGTGGAGTGAGAAGTC |
| PERP-F | ACAAGAATGACCCTCCTAATGC |
| PERP-R | GTCCAAACCTGTTGCCATAGT |
| STC2-F | GAATCCTGCGTGTGACATCC |
| STC2-R | GGTGAGCGAGGTAGCAAGA |
| CCL20-F | AACCATGTGCTGTACCAAGAG |
| CCL20-R | GGATGAAGAATACGGTCTGTGT |
| GDF15-F | TCGCTCCAGACCTATGATGAC |
| GDF15-R | GCTGTTTGGGCAGGAATCG |
| IGFBP4-F | GGAAGACTTGAAGCACAGAGG |
| IGFBP4-R | GCGAGCCACTGGAAGGATA |
| RHOB-F | TGTGCCTGTCCTAGAAGTGAA |
| RHOB-R | ACAAGTGTGGTCAGAATGCTAC |
| IGFBP3-F | CCAGCAAGCCATTCCTCCT |
| IGFBP3-R | AGAGTCAGCCTCCACATTCAG |
| ANKRD1-F | TGCCTTCCCTTGCTTCATTC |
| ANKRD1-R | TACTGCCACGCTACTGATATTC |
| CXCL5-F | ACCCAGGTTCTACTCTGTGAAA |
| CXCL5-R | AACAAGGAGAAGTTGTCCAAGG |
| HEXIM1-F | CAAGAAGAAGCGGCATTGGAA |
| HEXIM1-R | GAGGAACTGCGTGGTGTTATAG |
| INF2-M/AS-F | ACAAGTCCTTCTCCGAGGATGC |
| INF2-AS-R | AGGGGGAACCTCTTCCTGGCT |
| INF2-M-R | GGCCTGAGGCCTTCCTGGCT |
| LSM1-M/AS-F | TCTGCTGTTCCACCCTTTGT |
| LSM1-AS-R | GATCAATTTGGACTTGGAAA |
| LSM1-M-R | AGGAGAAATAGACTTGGAAA |
| LRP5-M/AS-F | CTCCCACCTGTGCCTGCTGT |
| LRP5-AS-R | GCACACGGAACTGCCTTACA |
| LRP5-M-R | CTCCTCGGCTCCTGCCTTACA |
| FLNA-M/AS-F | ACATCCACCTCTGAGCCATCA |
| FLNA-AS-R | TCACGGGCTAGGTGCTGGCAT |
| FLNA-M-R | ACTGTCACAGGTGCTGGCAT |
| LGMN-M/AS-F | CAGGAGGCAGCTCTTCAGTA |
| LGMN-AS-R | CGCTTCACAGGATAAAATTG |
| LGMN-M-R | GCATCTGGATGATAAAATTG |
| RPS27A-M-F | GCTTCTGGAAGTGGAGCCGC |
| RPS27A-AS-F | TCGCCTAAGGGGTGGAGCCGC |
| RPS27A-M/AS-R | GTCTTCCCCGTAAGGGTTTT |
